# Temperature-Dependent Conformational Dynamics of Substrate Entrance Loops in β-Glucosidase: Insights from Molecular Dynamics Simulations

**DOI:** 10.3390/ijms27104279

**Published:** 2026-05-11

**Authors:** Ki Hyun Nam

**Affiliations:** College of General Education, Kookmin University, Seoul 02707, Republic of Korea; structure@kookmin.ac.kr

**Keywords:** β-glucosidase, substrate entrance, conserved loop, flexibility, conformational change

## Abstract

β-Glucosidase (BGL) is widely used in biofuel production, industrial value-added chemicals, and food industry applications. The substrate entrance loops of BGL play a role in substrate specificity and accessibility. To better understand the substrate entrance loops of BGL, a high-resolution crystal structure of BGL from *Thermoanaerobacterium saccharolyticum* (TsaBGL) was determined at 1.65 Å, and all-atom molecular dynamics (MD) simulations were performed. The crystal structure of TsaBGL exhibited both folded and straight conformations of the flexible L3 loop, along with rigid conformations of L1, L2, and L4 loops. MD simulations revealed that the folded L3 loop transitioned to a straight conformation, indicating the preference for the straight conformation. At the optimal temperature for enzyme activity, the flexibility of the L3 loop of TsaBGL decreased, whereas that of the L1 loop increased. Moreover, the positions of L1 and L2 loops shifted in a direction opposite to the substrate entrance, resulting in an expanded substrate-binding entrance and increased substrate accessibility to the active site. MD simulations of three homologous BGLs showed that, despite sequence variability, a conserved dynamic trend exists in which the L1 loop exhibits higher flexibility, whereas the L3–L4 loops maintain structural rigidity under optimal conditions. These results provide both an understanding of the loop dynamics involved in substrate accessibility in BGLs and insights into enzyme engineering to improve catalytic performance.

## 1. Introduction

β-Glucosidases (BGLs) play a vital role in the final step of cellulose degradation by catalyzing the hydrolysis of β-1,4-glycosidic bonds in disaccharides or glucose-substituted polysaccharides, thereby releasing glucose [1,2,3]. BGLs are widely used in various biotechnological applications, including biofuel production [4,5], food processing [6,7], animal food [8,9], and biorefinery processes [10,11].

BGLs are classified into the glycoside hydrolase (GH) families GH1, GH3, GH5, GH9, GH30, and GH116 based on the amino acid sequence and structural similarity [12,13]. Among them, GH1 is an extensively investigated BGL that is widely used in biomass conversion and industrial applications [14,15,16]. GH1 BGLs adopt a conserved (β/α)_8_ TIM-barrel fold, and the substrate-binding site is located on the center of the barrel [3]. The substrate-binding pocket of BGLs is typically divided into three regions: the glycone site, aglycone site, and gatekeeper region [3,17]. The glycone site, located deep within the pocket, contains two conserved catalytic glutamate residues responsible for hydrolysis. The aglycone site contributes to substrate specificity and stabilization, and the gatekeeper region regulates substrate access to the active site [5,17].

The gatekeeper region of BGLs includes four loops (L1–L4) positioned above the substrate-binding pocket [18,19]. These four loop regions are involved in regulating substrate accessibility and enzyme specificity [18,19]. Consequently, engineering of the loop region of BGLs affects substrate accessibility and enzyme specificity [18,20,21]. For instance, in *Micrococcus antarcticus*, the L3 loop of β-glucosidase BglU is responsible for its psychrophilic adaptation and affects catalytic activity at low temperatures [22]. Furthermore, in *Cellulomonas biazotea*, glucose binding to the loop region within the gatekeeper region induces a stimulatory effect on enzyme activity [23]. Therefore, understanding the gatekeeper region of BGLs is essential for clarifying the substrate recognition mechanism and the unique molecular properties of BGLs. Although several studies have suggested that these loops undergo conformational changes during substrate recognition and binding [18,19], their molecular flexibility has not yet been completely elucidated.

The BGL from the hemicellulose-degrading *Thermoanaerobacterium saccharolyticum* (TsaBGL) exhibited maximum hydrolase activity at pH 6.0 and 55 °C [24]. It also exhibited glucose tolerance property, suggesting that it is attractive for industrial applications [24]. TsaBGL contains four loops (L1, L2, L3, and L4) involved in substrate recognition and accessibility above the substrate-binding pocket [25]. Structural analysis revealed that the L3 loop demonstrated significantly high flexibility, resulting in conformational changes of the substrate entrance, whereas the other three loops showed relatively rigid conformations [25]. These structural analyses provide insights into the substrate access mechanism mediated by the L3 loop conformation. Nevertheless, previously reported crystal structures of TsaBGL were collected under a cryogenic environment at 100 K, despite the optimal temperature for enzyme activity being 55 °C, which may provide biologically unreliable molecular flexibility. Because temperature is a crucial factor for enzyme activity, protein structure, and dynamics [26,27,28], understanding the functional conformations of the L1–L4 loop regions at optimal temperatures requires data collection of TsaBGL under optimal enzymatic conditions. Nonetheless, such studies remain technically challenging due to crystal instability at higher temperatures.

Therefore, in this study, to better understand the molecular flexibility of the substrate entrance region of TsaBGL, the high-resolution crystal structure of TsaBGL at 1.65 Å resolution was determined, and all-atom MD simulations of TsaBGL were performed at room temperature and optimal temperature for enzyme activity. The dynamics of the four loop regions and the substrate-binding entrance were examined. Moreover, additional MD simulations of three homologous BGLs were performed to confirm the conservation of the dynamics of the four loop regions at the gatekeeper region of BGL. The findings of this study provide both a better understanding of the MD of the substrate entrance region of BGL and insights into the substrate recognition mechanism of the BGL family.

## 2. Results

### 2.1. Structural Analysis of the Substrate Entrance Loop of TsaBgl

The high-resolution structure enables providing an accurate interpretation of the side chain conformation, hydrogen bond network, and water molecule interaction [29,30]. To apply a more accurate structural analysis and the more reliable model structure for MD simulation, the crystallographic study for TsaBGL was conducted to obtain the high-resolution structure. To date, two different crystal forms of TsaBGL have been reported, triclinic P1 and orthorhombic P2_1_2_1_2_1_, which contain four and one molecule in the asymmetric unit, respectively, and are grown under almost identical crystallization conditions [25].

Among them, four molecules of TsaBGL in the P1 crystal form exhibited two different conformations of the L3 loop, namely folded and straight conformations of the turn region [25]. To provide more information on loop flexibility, diffraction data for TsaBGL in the P1 crystal form were collected, and the structure was determined at 1.65 Å resolution, which is higher than that of previously determined P1 crystal forms at 1.90 and 2.10 Å resolution. The R_work_ and R_free_ values of the final model structure of TsaBGL determined in this study were 0.1953 and 0.2264, respectively (Table 1). The electron density map for all residues was clearly observed, except for Asn304 and Asp305 in the L3 loop of chain C.

**Figure 1:**
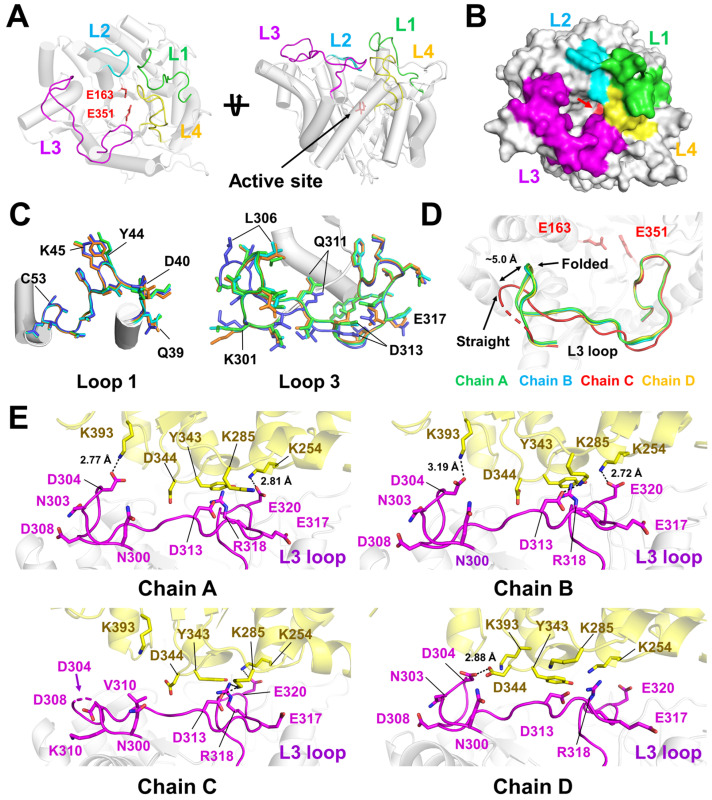
Crystal structure of TsaBGL. (**A**) Cartoon and (**B**) surface representations of TsaBGL. Active site is indicated by red arrow. The L1, L2, L3, and L4 loops are colored green, cyan, magenta, and yellow, respectively. (**C**) Superimposition of L1 and L3 loops in the four TsaBGL molecules in the asymmetric unit. (**D**) Close-up view of the superimposition of the folded (green for chain A, cyan for chain B, and yellow for chain D) and straight (red, chain C) conformations of the L3 loop of TsaBGL. (**E**) Interactions of the folded and straight conformations of the L3 loop of TsaBGL with a neighboring molecule (yellow) in the crystal packing.

The overall structure of the high-resolution TsaBGL is highly similar to that reported previously [24,25]. Briefly, the crystal structure of TsaBGL exhibits the typical TIM-barrel fold (Figure 1A). The active-site residues Glu183 and Glu351 are located inside the substrate-binding pocket. The positions of the four loops, L1 (Gln39–Asp54), L2 (Gly175–Asp183), L3 (Gln300–Tyr326), and L4 (Trp398–Ile416), have been defined previously [25] and are located above the substrate-binding cleft (Figure 1B). The L2 and L4 loops are directly involved in forming the substrate-binding pocket as gatekeeper regions, whereas the L1 loop is located above the L2 and L3 loops and is relatively distant from the substrate-binding pocket. The L3 loop is located away from the active site but contributes structurally to substrate access (Figure 1B).

Superimposition of the four TsaBGL molecules in the asymmetric unit revealed that the conformation of the L1 loop was similar, with a root-mean-square deviation (RMSD) of 0.088–0.217 Å. The subtle movement of the main chain between Arg38 and Gly41 was observed (Figure 1C). The conformations of the L2 and L4 loops among the four TsaBGL molecules were highly similar, with RMSD values of 0.056–0.096 and 0.054–0.088 Å, respectively (Supplementary Figure S1). The L3 loop exhibited significant conformational changes, with an RMSD of 0.112–0.826 Å, and adopts two distinct conformations with significant positional shifts (Figure 1D). In chains A, B, and D, the turn region (Lys301–Thr319) of the L3 loop was oriented to the substrate-binding pocket with folded conformation, whereas in chain C, it is directed toward the opposite direction in the straight conformation, consistent with previous reports [25]. The distance between the Cα atoms of Leu306 in the turn region of the L3 loop between the folded and straight conformations was ~5.07 Å (Figure 1D). Normalized B-factor analysis revealed that the four TsaBGL molecules exhibited relatively rigid L1, L2, and L4 loops, whereas the L3 loop displayed relatively high flexibility (Supplementary Figure S2).

Based on previous reports, although the L3 loop was expected to display conformational variability due to its flexibility, the conformation of the L3 loop in TsaBGL is largely similar to that in previously determined crystal structures of TsaBGL (PDB code 8WFT), despite subtle main-chain shifts being observed. Extended structural analysis revealed that the L3 loops of TsaBGL interact with neighboring molecules in the crystal packing. In chains A and B, the OD2 atom of Asp304 and the OE1 atom of Glu320 interacted with the NZ atoms of Lys393 and Lys254 from neighboring molecules at distances of 2.77–3.19 and 2.72–2.81 Å, respectively (Figure 1E). In chain D, the OD2 atom of Asp304 interacted with the OD2 atom of Asp344 from a neighboring molecule at a distance of 2.88 Å (Figure 1E). In contrast, in chain C, the electron density map for the Asp304 residue in the L3 loop was disordered, and the turn region of the L3 loop did not interact with neighboring molecules in the crystal packing (Figure 1E). However, the OD2 atom of Asp313, the NH2 atom of Arg318, and the OE1 atom of Glu320 in the straight conformation of the L3 loop interacted with the NZ atoms of Lys254 and Lys285 in neighboring molecules at distances of 2.59–3.44 Å in the crystal packing (Figure 1E), and the interaction area was larger than that of the folded L3 loop of TsaBGL in chains A, B, and D. These results indicate that the folded conformation of the L3 loop is less affected by crystal packing, except for the turn region.

Taken together, the observed conformation and flexibility of the L3 loop from the crystal structure may not accurately reflect its intrinsic flexibility due to crystal packing effects.

### 2.2. Molecular Dynamics Simulations of TsaBGL for Closed Conformation

The crystal structural analysis of BGL indicated that the molecular flexibility of TsaBGL may not be biologically relevant due to crystal packing effects. Moreover, the diffraction data of TsaBGL were collected at cryogenic temperature, which may result in biologically irrelevant molecular flexibility [31,32].

To better understand the molecular flexibility of the L1–L4 loops at the substrate entrance of TsaBGL, all-atom MD simulations were performed at 298 K and 328 K, corresponding to room temperature and optimal temperature for enzyme activity, respectively. Although structural analysis indicates that the folded conformation of the L3 loop represents a more reliable model due to reduced crystal packing effects, except for the turn region, MD simulations were conducted using both folded and straight conformations of the L3 loop of TsaBGL, TsaBGL^Folded-L3^, and TsaBGL^Straight-L3^, respectively, to capture diverse conformational dynamics.

**Figure 2:**
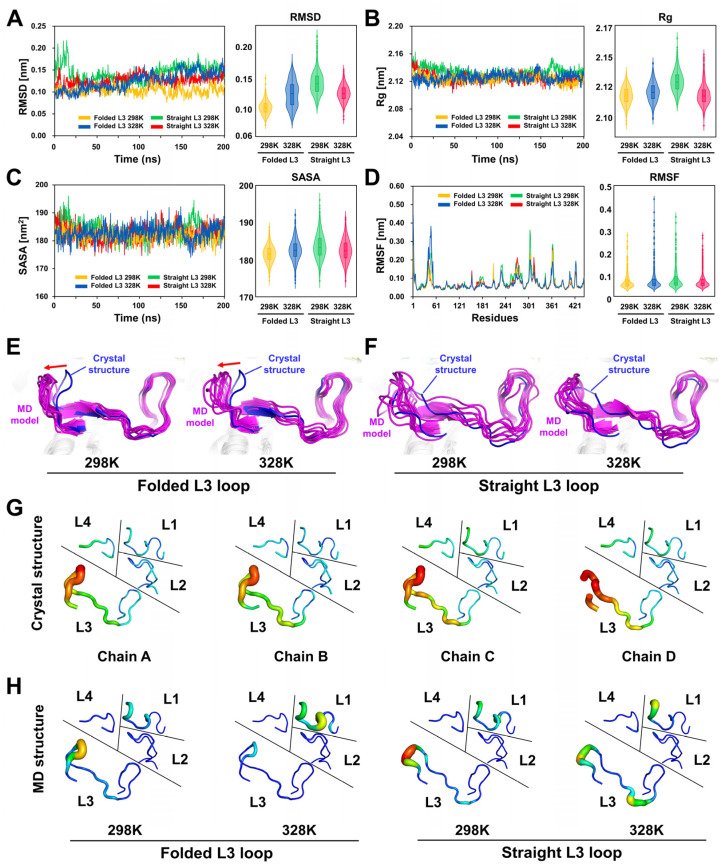
Analysis of the MD simulations of TsaBGL. (**A**) RMSD, (**B**) Rg, (**C**) SASA, and (**D**) RMSF plot obtained from MD simulations for TsaBGL^Folded-L3^ at 298 K (yellow), TsaBGL^Folded-L3^ at 328 K (blue), TsaBGL^Straight-L3^ at 298 K (green), and TsaBGL^Straight-L3^ at 328 K (red). (**E**) Analysis of the conformation ensemble of the (**E**) folded and (**F**) straight conformations of the L3 loop of TsaBGL from MD trajectories over time. (**G**) B-factor putty representation of the L1–L4 loops of the crystal structure of TsaBGL. (**H**) RMSF-derived B-factor putty representations of the L1–L4 loops of MD structure of TsaBGL^Folded-L3^ and TsaBGL^Straight-L3^ at 298 K and 328 K from MD trajectories.

The RMSD analysis revealed distinct temperature- and conformation-dependent behaviors. At 298 K, TsaBGL^Folded-L3^ remained relatively stable, with RMSD values of ~1.0 Å during the simulation (Figure 2A). In contrast, at 328 K, the RMSD of TsaBGL^Folded-L3^ gradually increased and reached ~1.5 Å at 200 ns. The average RMSD values of TsaBGL^Folded-L3^ at 298 K and 328 K were 1.03 and 1.25 Å, respectively. Furthermore, at 298 K, TsaBGL^Straight-L3^ initially exhibited a rapid increase in RMSD up to ~2.0 Å within the first ~25 ns. After this initial increase, the RMSD decreased to ~1.3 Å and gradually stabilized over time. Similarly, at 328 K, TsaBGL^Straight-L3^ exhibited an initial RMSD of ~1.5 Å within the first 10 ns, followed by stabilization within the range of ~1.0–1.2 Å. The average RMSD values of TsaBGL^Straight-L3^ at 298 K and 328 K were 1.42 and 1.26 Å, respectively (Figure 2A).

The radius of gyration (Rg) analysis showed that the initial Rg value of TsaBGL^Straight-L3^ at 298 K was slightly higher than that of other systems. Nevertheless, all systems converged to similar Rg values of ~21.2–21.3 Å at 200 ns (Figure 2B), indicating that the overall structural compactness was maintained irrespective of temperature or conformation. Similarly, the solvent-accessible surface area (SASA) remained stable across all systems, with average values ranging from 180 to 185 nm^2^ (Figure 2C).

In TsaBGL^Folded-L3^, L1 and L3 loops exhibited relatively high fluctuations at 298 K, with root-mean-square fluctuation (RMSF) values of approximately 2 and 3 Å, respectively (Figure 2D). At 328 K, the L1 loop showed a significant increase in fluctuation, with an RMSF of ~5 Å, whereas the L3 loop showed reduced fluctuation, with RMSF of ~1 Å (Figure 2D). In TsaBGL^Straight-L3^, the RMSF analysis revealed that L1 and L3 loops showed high fluctuations at 298 K, with maximum RMSF values of 2 and 3 Å, respectively, which were higher than those of L1 and L3 loops at 328 K, with the RMSF values being 1 and 2 Å, respectively (Figure 2D). The overall RMSD value of TsaBGL^Straight-L3^ at 298 K was higher than that at 328 K, which may be attributed to conformational changes in the loop regions rather than a direct temperature effect. Moreover, the L2 and L4 loops in both TsaBGL^Folded-L3^ and TsaBGL^Straight-L3^ exhibited no significant fluctuations, indicating a rigid conformation above the substrate-binding pocket.

The MD trajectory analysis showed that the turn region of the folded L3 loop in TsaBGL^Folded-L3^ gradually transitioned from a folded to a straight conformation over time (Figure 2E). In contrast, the remaining regions of the L3 loop, excluding the turn region, were similar between the crystal structure and the MD simulation. For TsaBGL^Straight-L3^, the L3 loop maintained its straight conformation throughout the MD simulation (Figure 2F). Nevertheless, the remaining regions of the L3 loop, excluding the turn region, exhibited significant conformational differences and positional shifts compared with the crystal structure. These findings suggest that the straight conformation of the L3 loop turn region is preferred, whereas the remaining L3 loop is more consistent with the folded conformation observed in the MD simulation of TsaBGL^Folded-L3^.

The B-factor putty representation of the crystal structure of TsaBGL showed that only the L3 loop displayed relatively high flexibility, whereas the other loops exhibited rigid conformations (Figure 2G). In the MD structure at 298 K, the flexibility of the L3 loop was relatively high and that of the L1 loop increased, whereas L2 and L4 loops were rigid, indicating that the overall flexibility of the four loop regions was similar to that of the crystal structure of TsaBGL, excluding the increased flexibility of the L1 loop (Figure 2H). In the MD model at 328 K, the flexibility of the L3 loop decreased, whereas that of the L1 loop increased (Figure 2H). The RMSF values of L1, L2, L3, and L4 loops at 298 K were approximately 0.93, 0.77, 1.07, and 0.62 Å, respectively, whereas at 328 K, the values were approximately 2.22, 0.68, 1.07, and 0.73 Å, respectively. These data indicate that the flexibility of L1 and L3 loops was affected by temperature factor.

### 2.3. Analysis of the Flexibility of L1–L4 Loops of TsaBGL

To explore how temperature-dependent loop flexibility affects the substrate entrance region of TsaBGL, MD models based on the folded L3 loop conformation at 298 and 328 K were further analyzed.

**Figure 3:**
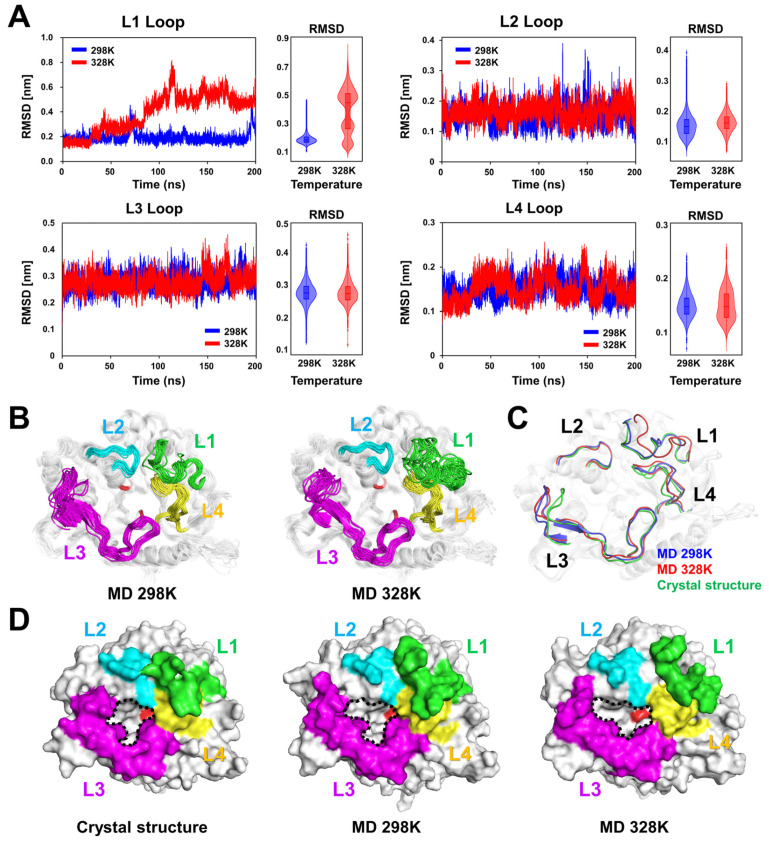
Analysis of L1–L4 loops from the MD trajectories of TsaBGL. (**A**) RMSD plot of L1–L4 loops from the MD simulations of TsaBGL at 298 K (blue) and 328 K (red) over 200 ns. (**B**) Dynamic ensemble of the L1–L4 loops of TsaBGL obtained from MD simulations over 200 ns. (**C**) Superimposition of the crystal structure of TsaBGL (green) with representative MD structures at 298 K (blue) and 328 K (red). (**D**) Surface structures of the crystal structure of TsaBGL and MD structures at 298 K and 328 K at 200 ns. The L1, L2, L3, and L4 loops are colored green, cyan, magenta, and yellow, respectively. The substrate entrance and active site are indicated by dotted lines and a red surface, respectively.

The RMSD analysis of the L1–L4 loop regions of TsaBGL indicated a significant increase in the RMSD value when the temperature was increased to the optimum for enzyme activity, whereas the other loop regions showed no significant change in RMSD (Figure 3A). The average RMSD values of the L1 loops of MD models at 298 K and 328 K were 1.92 and 3.92 Å, respectively, indicating an increase of approximately two-fold. The average RMSD values of the L2 loops of MD models at 298 K and 328 K were 1.52 and 1.62 Å, respectively, indicating a slight increase in the molecular flexibility of the L2 loop with increases in temperature. In contrast, the average RMSD values of the L3 (0.277 for both 298 K and 328 K) and L4 (0.149 for 298 K and 0.150 for 328 K) loops were highly similar (Figure 3A).

Structures extracted at regular intervals from the MD trajectories at 298 K exhibited subtle movement of the main-chain positions of the L1–L4 loop regions (Figure 3B), excluding the initial conformational change of the folded turn region of the L3 loop (Figure 2E). In the MD model at 328 K, subtle movement of the main chains of the L2–L4 loops was observed, whereas a significant conformational flexibility of the L1 loop was observed (Figure 3B). Furthermore, the position of the active site containing the two glutamate residues in MD models at 298 and 328 K was highly similar (Figure 3B).

Superimposition of the representative MD structures of TsaBGL at 298 K and 328 K extracted by the RMSD-based clustering of the production trajectories revealed an overall similarity, with the RMSD being 0.691 Å; however, the position of the L1 loop was significantly different (Figure 3C). The overall position of the L1 loop of the MD structure at 298 K was similar to that of the L1 loop of the crystal structure (Figure 3C), whereas the position of the L1 loop of the MD structure at 328 K shifted to the opposite direction of the substrate-binding pocket (Figure 3C). The shift range of the Cα atoms between the L1 loops of representative MD models at 298 and 328 K was 0.59–6.55 Å (average distance, 3.64 Å). Subtle movements of the main chains in L2, L3, and L4 loops were also observed, with Cα shift ranges of 0.36–0.89 Å (average, 0.63 Å), 0.25–2.87 Å (average, 1.08 Å), and 0.28–1.98 Å (average, 0.81 Å), respectively.

To understand how the position shifts of L1–L3 loops result in different architectures of the substrate-binding entrance region of TsaBGL, surface structures of the crystal structure of TsaBGL and the MD structures at 298 K and 328 K obtained from the trajectory at 200 ns were examined. Results showed that the architecture of the substrate entrance region between the crystal structure and MD structure at 298 K was similar, excluding the L3 loop region (Figure 3D). These surface structures exhibited partial hindrance of the active site region in the top view of the TIM-barrel domain. In the MD structure at 323 K, the L1 and L2 loop regions shifted to the opposite direction of the active-site region, which increased the volume of the substrate-accessible region, including that of the gatekeeper region (Figure 3D). In particular, the active site of TsaBGL was clearly observed in the top view of the TIM-barrel domain (Figure 3D). These MD data indicated that the substrate accessibility increases at the optimal temperature. The calculated cavity volumes of the substrate-binding pocket and substrate access region in the crystal structure and MD models at 298 K and 328 K were approximately 996, 1753, and 2150 Å^3^, respectively. These findings indicate that temperature-dependent conformational changes in the L1–L3 loops of TsaBGL can affect substrate accessibility.

### 2.4. MD Analysis of the L1–L4 Loop Region of Homologous BGLs

**Figure 4:**
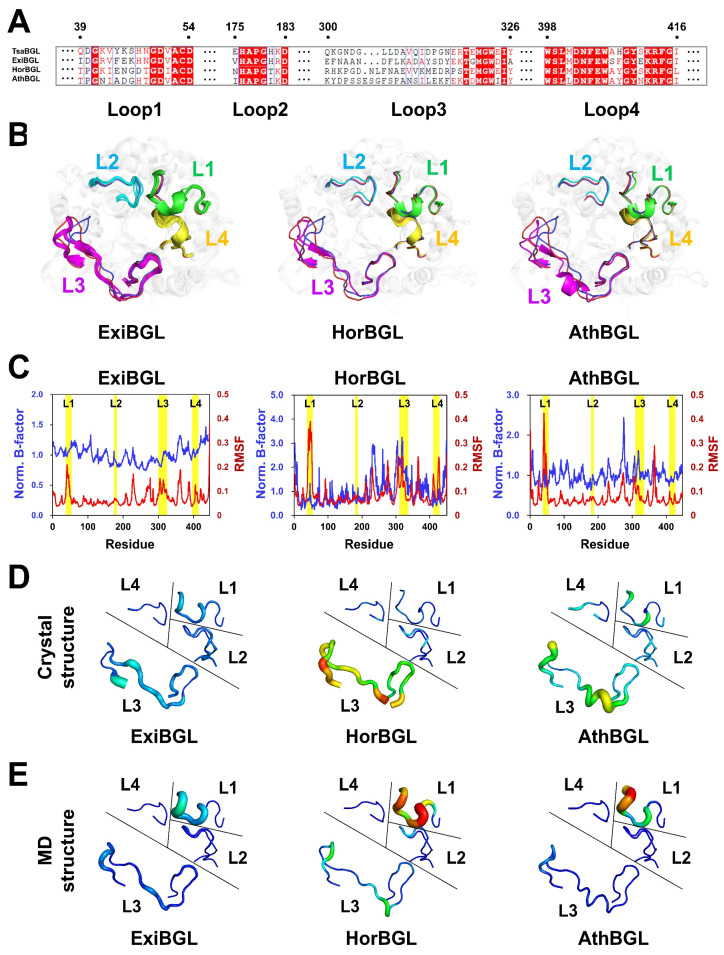
Amino acid and structure analysis of TsaBGL homologs. (**A**) Partial amino acid sequence alignment of TsaBGL (UniProt: I3VXG7) with BGL from *Exiguobacterium* sp. (ExiBGL, C4L1S4), *H. orenii* (HorBGL, B8CYA8), and *A. thermocellus* (AthBGL, P26208). (**B**) Superposition of TsaBGL (chain D, green) with ExiBGL (PDB code: 6WIU, cyan), HorBGL (3TA9, magenta), and AthBGL (9UPT, yellow). Sixteen ExiBGL and two HorBGL molecules from the asymmetric unit were superimposed. (**C**) Average B-factor values (blue) from the crystal structures and RMSF values (red) obtained from the MD simulations of ExiBGL, HorBGL, and AthBGL. (**D**) B-factor putty representations of the crystal structures of ExiBGL (chain A), HorBGL (chain A), and AthBGL, collected at cryogenic temperature. (**E**) B-factor putty representations derived from the RMSF values of ExiBGL, HorBGL, and AthBGL obtained from the MD simulations at their optimal temperatures for enzyme activity.

To determine whether the flexibility of L1–L4 loops in TsaBGL is conserved among BGLs, amino acid sequence analysis and MD simulations of homologous BGLs were performed (Table S1). Homologous BGL search showed that TsaBGL exhibits high amino acid sequence similarity (>87.2%) with those of BGLs from *Thermoanaerobacterium aotearoense* (TaoBGL), *Thermoanaerobacterium thermosaccharolyticum* (TthBGL), and *Thermoanaerobacterium butyriciformans* (TbuBGL). These homologous BGLs exhibit highly conserved sequences across L1–L4 loop regions (Supplementary Figure S3). Furthermore, BGLs from *Thermoanaerobacter uzonensis* (TuzBGL), *Thermoanaerobacter pseudethanolicus* (TpsBGL), *Thermoanaerobacter thermohydrosulfuricus* (TthBGL2), *Thermoanaerobacter brockii* subsp. finnii (TbrBGL and TbrBGL2), *Thermoanaerobacter siderophilus* SR4 (TsiBGL), *Thermoanaerobacter wiegelii* Rt8.B1 (TwiBGL and TwiBGL2), *Thermoanaerobacter mathranii* subsp. mathranii (TmaBGL), *Thermoanaerobacter pseudethanolicus* (TpsBGL), *Caldanaerobacter subterraneus* subsp. tengcongensis (CsuBGL), *Thermoanaerobacter italicus* (TitBGL), *Thermoanaerobacter pentosaceus* (TpeBGL), *Thermoanaerobacterium saccharolyticum* (TsaBGL2), *Thermoanaerobacterium aotearoense* SCUT27 (TaoBGL2), and *Thermoanaerobacterium xylanolyticum* (TxyBGL) with moderate sequence similarity (63.1–67.7%) exhibited high similarity in the L2 and L4 loops, whereas the L1 and L3 loops exhibited relatively low sequence similarity (Supplementary Figure S3). In addition, BGLs from *Exiguobacterium* sp. (ExiBGL), *Halothermothrix orenii* (HorBGL), and *Acetivibrio thermocellus* (AthBGL) exhibited relatively low sequence similarity (50.7–54.1%) with partial conservation in the L4 loop, whereas the other loop regions exhibited poor conservation (Figure 4A). Moreover, the L3 loops of HorBGL and AthBGL are longer than that of TsaBGL by two and three residues, respectively. These results indicate that the amino acid sequences and lengths of the loop regions above the substrate-binding pocket in the BGL family are diverse.

Among these BGL homologs, studies have determined the crystal structures of ExiBGL (PDB code: 6WIU) [33], HorBGL (3TA9) [34], and AthBGL (9UPT) [35]. To determine whether the structural properties of the molecular flexibility of L1–L4 loops in TsaBGL are conserved among BGL homologs, the molecular flexibility of the L1–L4 loops of ExiBGL, HorBGL, and AthBGL was examined (Supplementary Figure S4).

In the crystal structure of ExiBGL, 16 molecules are present in the asymmetric unit. Superimposition of these molecules shows that the overall conformations of L1–L4 loops are highly similar, although there were slight differences in the main-chain positions (Figure 4B). Superimposition of TsaBGL and ExiBGL revealed similarity, with an RMSD value of 0.617–0.837 Å, and the conformations of L1, L2, and L4 loops were similar, whereas the L3 loop adopted a different conformation (Figure 4B). Regarding HorBGL, two molecules occupied in the asymmetric unit. The entire L3 loop of HorBGL was not modeled in the structure due to missing residues (Glu319 in chain A; Gly206, Asp307, and Glu319 in chain B), indicating that the L3 loop is flexible. Superimposition of TsaBGL and HorBGL revealed similarity, with an RMSD value of 0.542–0.550 Å, and showed that the conformations of L1, L2, and L4 loops were similar, whereas the L3 loop adopted a different conformation (Figure 4B). The crystal structure of AthBGL contained one molecule in the asymmetric unit. Superimposition of TsaBGL and HorBGL showed similarity, with an RMSD value of 0.672 Å, and shows positional shifts in all loop regions, with a distinct conformation of the L3 loop when superimposed with TsaBGL (Figure 4B).

The B-factor values of the L1–L4 loops of ExiBGL varied depending on the molecule and were slightly higher than those of the TIM-barrel core (Supplementary Figure S5). The average B-factor analysis revealed no significant molecular flexibility in the L1–L4 loop regions of ExiBGL (Figure 4C,D). In HorBGL, the L1, L2, and L4 loops appeared relatively rigid, whereas the L3 loop showed significantly higher flexibility (Figure 4C,D and Supplementary Figure S5). In AthBGL, the overall B-factor values of L1–L4 loops were relatively low, with partial flexibility observed in the L3 loop (Figure 4C,D and Supplementary Figure S5). These findings indicate that the molecular flexibility of L1–L4 loops varies among BGLs.

The crystal structures of ExiBGL, HorBGL, and AthBGL have been determined at cryogenic temperature [32,33,34]. To better understand the molecular flexibility of the L1–L4 loops of these homologous BGLs, MD simulations were performed for ExiBGL, HorBGL, and AthBGL at their optimal temperatures for enzyme activity at 318 K, 343 K, and 338 K, respectively. Results showed that the RMSD of ExiBGL and AthBGL remained stable during the MD simulation, whereas the RMSD of HorBGL gradually increased and then stabilized (Supplementary Figure S6). No significant changes were found in Rg and SASA values for ExiBGL, HorBGL, and AthBGL, indicating that the overall BGL structures remained stable (Supplementary Figure S6). The RMSF analysis showed that the L1 loop regions of ExiBGL, HorBGL, and AthBGL significantly increased when compared with the B-factor values from the crystal structures (Figure 4C). The RMSF of the L3 loop of ExiBGL slightly increased from that of the rigid conformation, whereas the RMSF of the L3 loops of HorBGL and AthBGL decreased compared with the B-factor values of the crystal structures (Figure 4C,E). Moreover, the RMSF values of L2 and L4 loops showed no significant changes compared with the B-factor values of their crystal structures (Figure 4C,E). The calculated cavity volumes of the substrate-binding pockets in the crystal structures of ExiBGL, HorBGL, and AthBGL were 612, 2257, and 1040 Å^3^, respectively, whereas those in the MD structures were 763, 1790, and 2502 Å^3^, respectively. Remarkably, ExiBGL and AthBGL showed an increase in cavity volume with increasing temperature, similar to that of TsaBGL, whereas HorBGL exhibited a decrease. These results indicate that increasing the temperature does not necessarily result in an expansion of the substrate-binding pocket.

## 3. Discussion

BGL is essential for the final step of cellulose degradation in biomass conversion and is widely applied in various biotechnological fields, including biofuel production and the food industry. Understanding the structural properties of the substrate-binding entrance is important for clarifying its molecular mechanism and provides insights into protein engineering for improved enzyme activity.

The substrate entrance loops of BGL are essential for substrate selectivity and accessibility [19,36]. Previous comparative analyses of the amino acid sequences and structures of 46 BGL proteins revealed that L2 and L3 loops are diverse [25]. The crystal structure analysis of TsaBGL indicated that the conformation of the L3 loop is critical for the shape of the substrate-binding entrance [25]. Although this observation remains valid, extended structural analysis of TsaBGL in the present study revealed that the conformation and flexibility of the L3 loop observed in the crystal structure may not be accurate due to crystal packing effects. Moreover, as the X-ray diffraction data for the crystal structure were collected under cryogenic conditions, the observed molecular flexibility may not accurately reflect the native state.

To address this issue, MD simulations of TsaBGL were performed at room temperature and at the optimal temperature for enzyme activity. The results of MD simulation revealed that the folded L3 loop of TsaBGL transitioned to a straight conformation, suggesting the preference for the straight conformation of the L3 loop. These results suggest that if the loop or other regions of interest observed in the crystal structure are involved in crystal packing, their conformations and apparent flexibility are influenced by packing interactions. Therefore, when analyzing loop conformations at the substrate entrance of BGL, it is important to consider the potential involvement in crystal packing and to complement structural observations with MD simulations to identify more stable conformations.

The flexibility of the L1 and L3 loops of TsaBGL observed in the MD structure at 298 K was comparable to that in the crystal structure. Nevertheless, at the optimal temperature, the flexibility of the L1 loop increased, whereas that of the L3 loop decreased compared with both the crystal structure and MD structure at 298 K. The increased flexibility of the L1 loop, along with its displacement away from the substrate entrance, resulted in an expansion of the accessible volume of the substrate-binding region. Moreover, a positional shift of the L2 loop further enlarged the opening of the substrate-binding pocket, as observed in the top view of the TIM-barrel domain. These findings suggest that temperature-dependent changes in loop flexibility and positioning modulate the architecture of the substrate-binding entrance in TsaBGL through a conformational selection mechanism, thereby regulating substrate accessibility.

The structural analyses of ExiBGL, HorBGL, and AthBGL revealed differences in amino acid sequences for L1, L2, and L3 loops, as well as differences in molecular flexibility and the architecture of the substrate entrance region. However, the MD simulations showed that all these BGLs exhibited relatively high flexibility in the L1 loop region, whereas the L3 loop remained relatively rigid at their optimal temperatures for enzyme activity. These MD results for BGL homologs are consistent with those of TsaBGL at its optimal temperature, indicating a similar trend in the molecular flexibility of L1–L4 loops under optimal conditions despite differences in amino acid sequence and length.

The observed differences in the conformation and positioning of L1–L4 loops between the crystal and MD structures of TsaBGL provide complementary insights into the molecular mechanisms and engineering of BGLs. Analysis of the crystal structure of TsaBGL suggests that the rigid conformations of L1, L2, and L4 loops, together with the high flexibility of the L3 loop, affect the architecture of the substrate-binding entrance depending on the conformation of the L3 loop, thus emphasizing its potential as a target for structural stabilization or engineering. Conversely, the MD-based analysis of TsaBGL indicated that L2–L4 loops maintained relatively rigid conformations and did not directly alter the architecture of the substrate-binding pocket entrance.

Instead, substrate accessibility was primarily affected by the flexibility of the L1 loop, suggesting its role as a key determinant of dynamic substrate access. Therefore, a comprehensive structural analysis integrating both crystal structures and MD simulations is required to gain a complete understanding of the relationship between loop conformation and substrate accessibility in BGLs. In particular, site-directed mutagenesis of key residues in L1 and L3 loops will be required to validate their distinct roles in substrate accessibility and selectivity.

Previous research based on crystal structures reported that the highly flexible L3 loop of TsaBGL modulates the architecture of the substrate-binding entrance [25]. In contrast, the combined analysis of crystal structures and molecular dynamics (MD) simulations in this study revealed that not only the L3 loop but also the L1 and L2 loops can structurally impact the substrate access region. In particular, the flexibility of L1 and L3 loops was modulated in a temperature-dependent manner. These results extend beyond the limitations of static crystal structures and provide new insights into loop-mediated substrate accessibility under optimal conditions.

The results of the MD simulations of TsaBGL, ExiBGL, HorBGL, and AthBGL indicated that L2–L4 loops exhibited rigid conformations at the optimal temperature for enzyme activity, demonstrating that the substrate entrance region is relatively rigid under these conditions and that this rigid architecture may contribute to substrate selectivity. Conversely, the L1 loop is not directly involved in the substrate entrance region and is therefore considered to contribute more to substrate accessibility than to substrate selectivity. Accordingly, based on the MD simulations, the L2–L4 loops and the L1 loop may serve as potential targets for protein engineering to improve the substrate selectivity and accessibility of BGL, respectively; however, no experimental evidence is provided in this study. Therefore, further biochemical studies, including mutagenesis, will be required to more accurately confirm the molecular functions of the L1–L4 loops.

Meanwhile, this study suggests that the expansion of the cavity volume of the substrate-binding pocket may be associated with increased substrate accessibility based on structural analysis; however, no experimental evidence is presented in this study. To verify the relationship between cavity volume and substrate accessibility, further studies, including mutagenesis, as well as analyses of specific activity, catalytic efficiency, or substrate preference, are required.

Temperature is a vital factor that affects enzyme activity and protein structure [37,38,39]. Crystal structures collected at cryogenic temperatures may exhibit biologically irrelevant low molecular flexibility compared with those collected at room temperature [32,40]. For instance, in xylanase TsaGH11, the thumb domain involved in substrate recognition is clearly observed in the crystal structure collected at cryogenic temperature, whereas this region is disordered in the crystal structure collected at room temperature obtained by serial synchrotron crystallography [41]. Similarly, structures of lysozyme collected at room temperature show that both the overall structure and loop regions involved in substrate recognition exhibit relatively higher flexibility than cryogenic structures [42]. Meanwhile, structures collected at room temperature obtained using conventional macromolecular crystallography with synchrotron radiation may still exhibit biologically irrelevant flexibility due to radiation damage [43,44,45]. To obtain more accurate structural flexibility of BGLs, time-resolved serial crystallography using the mix-and-inject approach may be a useful approach [46,47,48,49], as it enables the determination of structures collected at room temperature and also minimizes radiation damage.

In conclusion, this study reports the temperature-dependent conformational changes and flexibility of the substrate entrance loops of BGLs based on crystal structures and MD simulations. This study provides a better understanding of the molecular properties of the substrate entrance region of BGLs at optimal temperatures and also provides insights into BGL engineering for efficient future industrial applications.

## 4. Materials and Methods

### 4.1. Protein Preparation and Crystallization

The protein expression and purification and crystallization procedures were performed as reported previously [24]. Briefly, the recombinant TsaBGL was overexpressed in *Escherichia coli* BL21(DE3) by adding the isopropyl β-d-1-thiogalactopyranoside. The proteins were purified by Ni-NTA affinity chromatography and size exclusion chromatography. The purity of the proteins was confirmed by SDS-PAGE. Crystallization was performed using the hanging drop vapor diffusion method at 20 °C. The protein solution (30 mg/mL, 2 μL) was mixed with the crystallization solution (2 μL) containing 0.1 M Tris–HCl, pH 7.0, 25–30% (*w*/*v*) polyethylene glycol 3350, and 0.2 M MgCl_2_ and equilibrated against the crystallization solution (500 μL). Suitable crystals for X-ray diffraction experiment were obtained within 2 weeks.

### 4.2. Data Collection

X-ray diffraction data were collected at beamline 11C of the Pohang Light Source II (PLS-II, Pohang, Republic of Korea) [50]. The TsaBGL crystal was soaked in a cryoprotectant solution containing the crystallization solution supplemented with 20% (*w*/*v*) ethylene glycol for 10 s. Diffraction data were collected under a liquid-nitrogen stream at 100 K and recorded on a PILATUS 6M detector (DECTRIS, Baden, Switzerland). Diffraction data were processed with Xia2 [51] at the global science data hub center (GSDC) at Korea Institute of Science and Technology Information (KISTI, Daejeon, Republic of Korea) [52].

### 4.3. Structure Determination

The phase problem was solved by molecular replacement with MOLREP [53] implemented in CCP4 [54]. The crystal structure of native TsaBGL (PDB code: 8WFT) [25] was used as a search model. Model building was performed using COOT [55]. The structure refinement was performed using phenix.refine implemented in PHENIX [56]. The final model structure was validated using MolProbity [57].

### 4.4. Molecular Dynamics Simulations

The crystal structure of the L3 folded and straight conformations of TsaBGL determined in this study were used as the initial model for MD simulations. All-atom MD simulations were performed using GROMACS [58]. AMBER99SB-ILDN force field [59] was applied for the system, which was solvated in a triclinic box filled with TIP3P [60] water molecules. The minimum distance between protein surface and the box boundary was 10 Å. The system charge was neutralized by adding counterions. Energy minimization of the model system was applied using the steepest descent algorithm until the maximum force was <1000 kJ mol^−1^ nm^−1^. The system was then equilibrated by NVT equilibration for 100 ps at the target temperature using a V-rescale thermostat. Further NPT equilibration was applied for 100 ps using a Parrinello–Rahman barostat to maintain a pressure of 1 bar. The MD simulations for all systems were performed at 298 K and 328 K for 200 ns.

The model coordinates of ExiBGL (PDB code: 6WIU, chain A), HorBGL (3TA9, chain A), and AthBGL (9UPT) were obtained from Protein Data Bank [61]. In the crystal structure of HorBGL, the Ser333 residue was not modeled due to a lack of electron density. It was manually added for MD simulations with corrected geometry based on the Ramachandran plot. All-atom MD simulations for ExiBGL, HorBGL, and AthBGL were conducted under the same conditions as those for TsaBGL at temperatures of 318 K, 343 K, and 338 K, respectively.

### 4.5. Trajectory Analysis

The RMSD of Cα atoms from the MD trajectories relative to the crystal structure, Rg, SASA, and RMSF of the MD trajectories were analyzed using GROMACS tools and in-house scripts. Representative structures of TsaBGL for each system were extracted by RMSD-based clustering of the production trajectories using a cutoff of 2.0 Å.

### 4.6. Bioinformatics

Homologous proteins were searched using BLAST [62]. Alignment of amino acid sequences was performed using Clustal Omega [63] and visualized using ESPript 3.0 [64]. The cavity volume of the substrate-binding pocket was calculated using CB-DocK2 [65]. Structure figures were visualized using PyMOL (Schrödinger, LLC). 

## Figures and Tables

**Table 1:** Data collection and structure refinement statistics of TsaBgl.

Data collection	TsaBgl		
Space group	P1		
Unit cell (Å)			
a, b, c	63.12, 72.53, 97.19		
α, β, γ	92.56, 91.28, 95.16		
	Overall	Low	High
Resolution (Å)	72.40–1.65	72.40–4.47	1.68–1.65
Total observations	682,547	36,053	32,116
Unique observations	183,242	9499	8841
Completeness (%)	87.9	91.0	85.0
Multiplicity	3.7	3.8	3.6
I/sigma	5.6	13.7	1.1
CC1/2	0.992	0.996	0.371
Structure refinement			
Resolution (Å)	72.40–1.65 (1.67–1.65)		
R_work_	0.1953 (0.3524)		
R_free_	0.2264 (0.3509)		
R.m.s. deviations			
Bonds (Å)	0.003		
Angles (°)	0.665		
*B* factors (Å^2^)			
Protein	21.93		
Water	31.55		
Ramachandran plot (%)			
Favored	96.99		
Allowed	3.01		
Disallowed	0.00		
PDB code	25PT		

Values for the outer shell are given in parentheses.

## Data Availability

The structure factor and coordinates were deposited at the Protein Data Bank (https://www.rcsb.org) under access code 25PT. The data that support the findings of this study are available on request from the corresponding author.

## Supplementary Materials

The following supporting information can be downloaded at: https://www.mdpi.com/article/10.3390/ijms27104279/s1.
