# Supplementary material for: Temperature-Dependent Conformational Dynamics of Substrate Entrance Loops in β-Glucosidase: Insights from Molecular Dynamics Simulations
Source: Int J Mol Sci. 2026 May 11;27(10):4279. doi: 10.3390/ijms27104279 (PMC13207776; doi:10.3390/ijms27104279)
Supplement: Supplementary file 1 [file ijms-27-04279-s001.zip › ijms-4288064-supplementary.pdf]

Supplementary Data

Temperature-Dependent Conformational Dynamics of Substrate Entrance Loops in β-Glucosidase: Insights from Molecular Dynamics Simulations

Ki Hyun Nam

College of General Education, Kookmin University, Seoul 02707, Republic of Korea; structure@kookmin.ac.kr

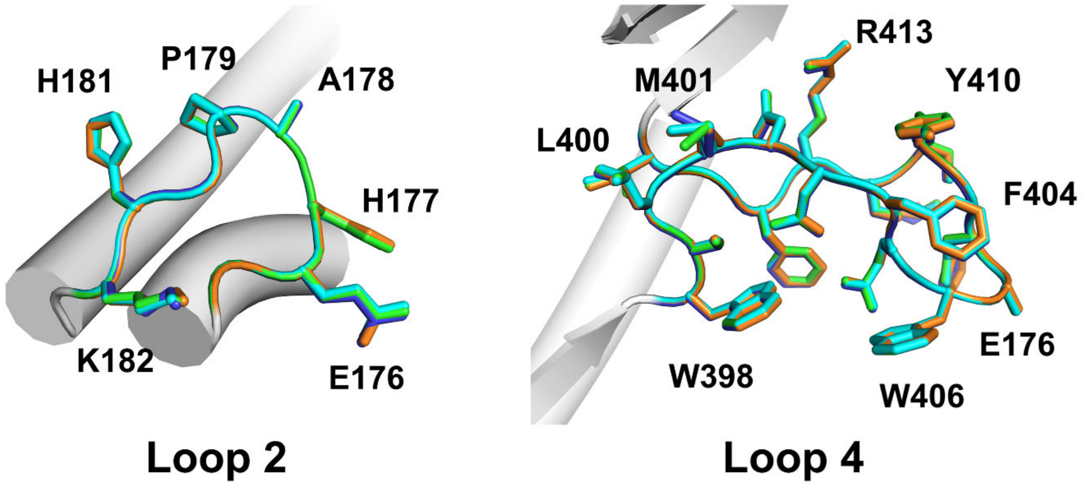

Supplementary Figure S1. Superimposition of L2 and L4 loops of the four TsaBGL molecules in the asymmetric unit. Chains A, B, C, and D are colored green, cyan, orange, and blue, respectively.

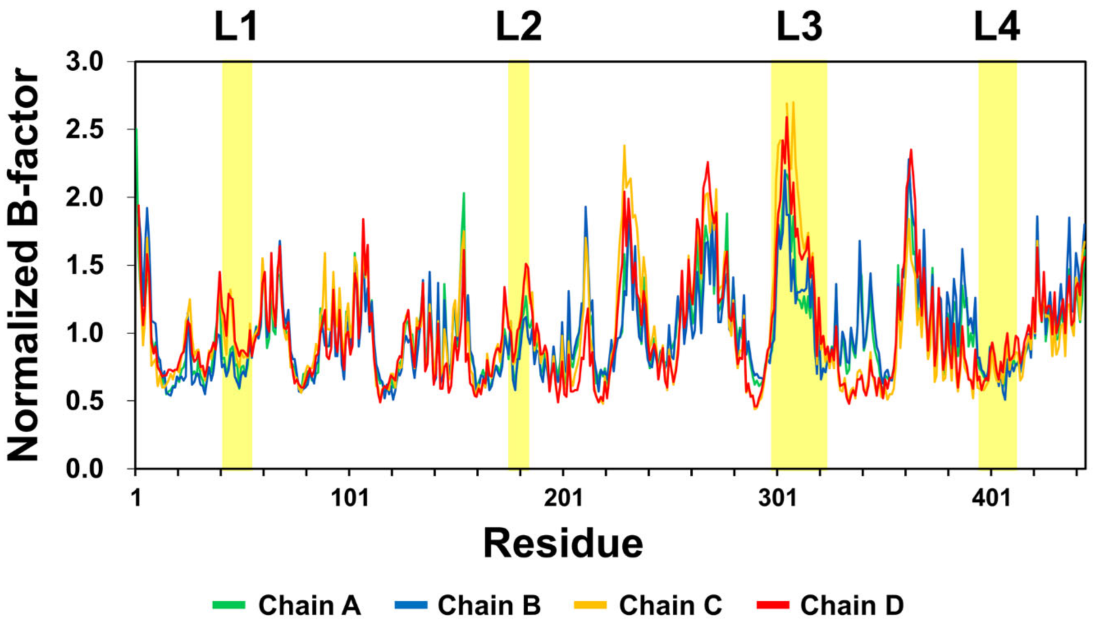

Supplementary Figure S2. B-factor profiles of the four TsaBGL molecules in the asymmetric unit. The L1–L4 loop regions are highlighted in yellow.

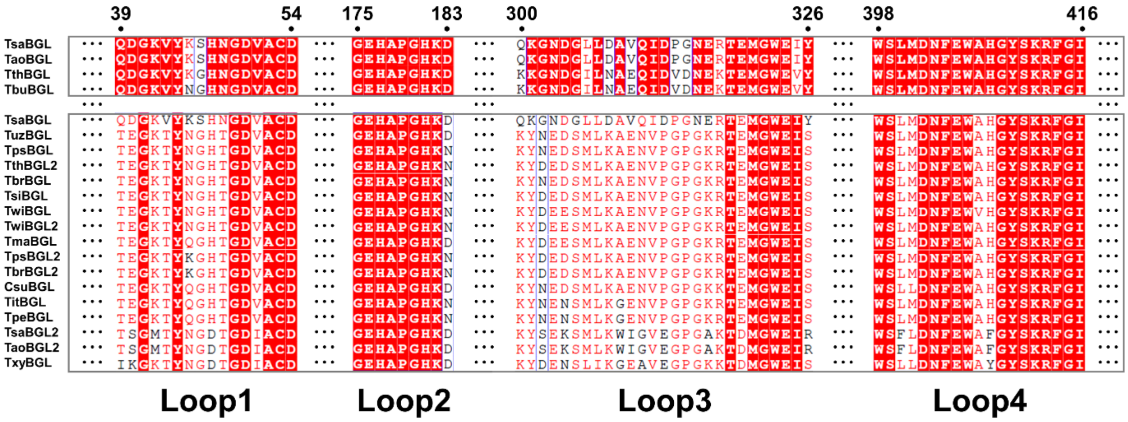

Supplementary Figure S3. Amino acid analysis of TsaBGL homologs. Partial amino acid sequence alignment of TsaBGL (UniProt: I3VXG7) with BGLs obtained from *T. aotearoense* (TaoBGL, W9EAC3), *T. thermosaccharolyticum* (TthBGL, D9TR57 and TthBGL2, M8CYT6), *T. butyriciformans* (TbuBGL, A0ABS4NH26), *T. uzonensis* DSM 18761 (TuzBGL, A0A1M4SU08), *T. pseudethanolicus* (TpsBGL, B0KCV1), *T. brockii* subsp. finnii (TbrBGL, E8UQS3; TbrBGL2, E8URA9), *T. siderophilus* SR4 (TsiBGL, I8R1J8), *T. wiegelii* Rt8.B1 (TwiBGL, G2MRY3 and TwiBGL2, G2MUZ4), *T. mathranii* subsp. mathranii (TmaBGL, A0ABM5LN57), *T. pseudethanolicus* (TpsBGL, B0KDF9), *C. subterraneus* subsp. tengcongensis (CsuBGL, Q8RCQ8), *T. italicus* (TitBGL, D3T6M2), *T. pentosaceus* (TpeBGL, A0ABT9M0X5), *T. saccharolyticum* (TsaBGL2, I3VS74), *T. aotearoense* (TaoBGL2, W9EEC0), and *T. xylanolyticum* (TxyBGL, F6BL86).

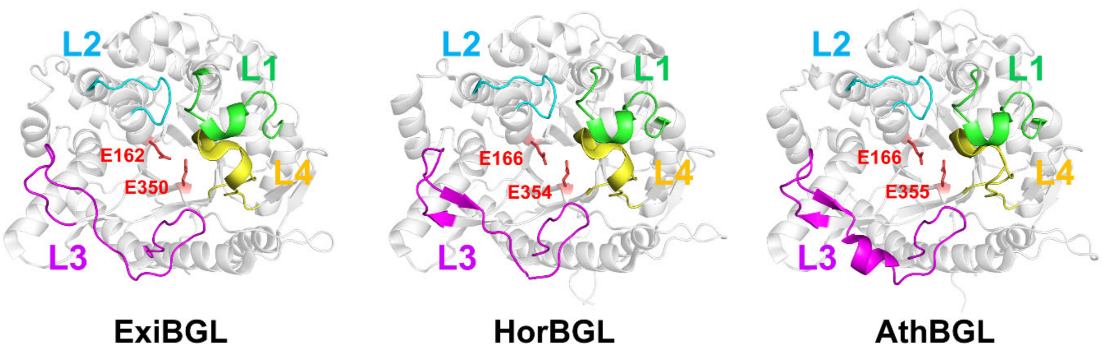

Supplementary Figure S4. Cartoon representation of ExiBGL (PDB code: 6WIU), HorBGL (3TA9), and AthBGL (9UPT). The L1, L2, L3, and L4 loops are colored green, cyan, magenta, and yellow, respectively.

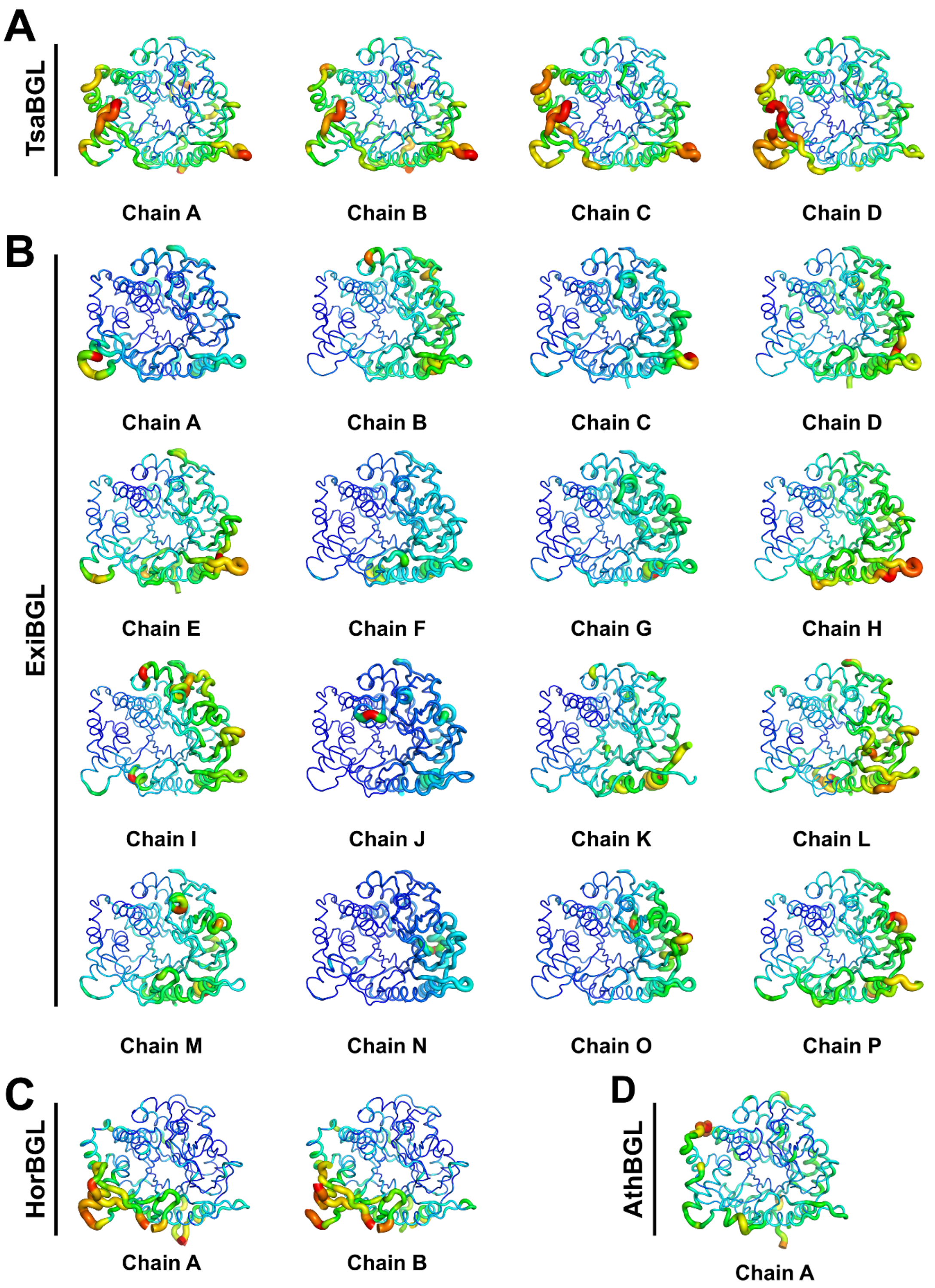

Supplementary Figure S5. B-factor putty representations of ExiBGL (PDB code: 6WIU), HorBGL (3TA9), and AthBGL (9UPT). Sixteen ExiBGL and two HorBGL molecules are present in the asymmetric unit.

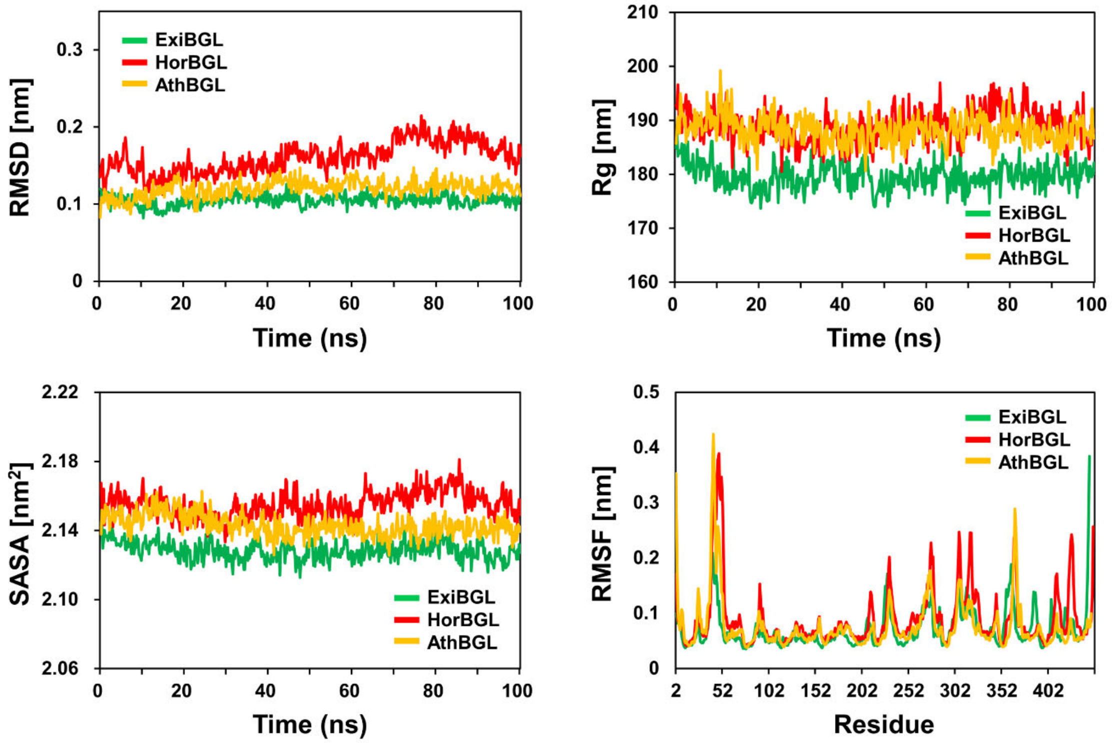

Supplementary Figure S6. MD simulations of ExiBGL, HorBGL, and AthBGL. Analysis of RMSD, Rg, SASA, and RMSF for ExiBGL (green), HorBGL (red), and AthBGL (yellow).

Table S1. Summary of TsaBGL homologs.

UniProt Code	Protein name	Organism	Length (AA)	Sequence identity (%)	Alignment score
I3VXG7	β-glucosidase	*Thermoanaerobacterium saccharolyticum* (strain DSM 8691 / JW/SL-YS485)	444	100	2410
W9EAC3	β-glucosidase	*Thermoanaerobacterium aotearoense* SCUT27	444	100	2410
D9TR57	β-glucosidase	*Thermoanaerobacterium thermosaccharolyticum* (strain ATCC 7956 / DSM 571 / NCIMB 9385 / NCA 3814 / NCTC 13789 / WDCM 00135 / 2032) (*Clostridium thermosaccharolyticum*)	444	87.2	2164
A0ABS4NH26	β-glucosidase	*Thermoanaerobacterium butyriciformans*	444	87.2	2163
A0A1M4SU08	β-glucosidase	*Thermoanaerobacter uzonensis* DSM 18761	447	67.7	1682
B0KCV1	β-glucosidase	*Thermoanaerobacter pseudethanolicus* (strain ATCC 33223 / 39E) (Clostridium thermohydrosulfuricum)	446	67.5	1681
M8CYT6	β-glucosidase	*Thermoanaerobacter thermohydrosulfuricus* WC1	446	67.5	1681
E8UQS3	β-glucosidase	*Thermoanaerobacter brockii* subsp. finnii (strain ATCC 43586 / DSM 3389 / AKO-1) (Thermoanaerobacter finnii)	446	67.5	1681
I8R1J8	β-glucosidase	*Thermoanaerobacter siderophilus* SR4	446	67.3	1680
G2MRY3	β-glucosidase	*Thermoanaerobacter wiegelii* Rt8.B1	447	66.6	1663
G2MUZ4	β-glucosidase	*Thermoanaerobacter wiegelii* Rt8.B1	447	66.6	1660
A0ABM5LN57	β-glucosidase	*Thermoanaerobacter mathranii* subsp. mathranii (strain DSM 11426 / CCUG 53645 / CIP 108742 / A3)	447	66.1	1659
B0KDF9	β-glucosidase	*Thermoanaerobacter pseudethanolicus* (strain ATCC 33223 / 39E) (*Clostridium thermohydrosulfuricum*)	447	67.1	1657
E8URA9	β-glucosidase	*Thermoanaerobacter brockii* subsp. finnii (strain ATCC 43586 / DSM 3389 / AKO-1) (*Thermoanaerobacter finnii*)	447	67.1	1657

Q8RCQ8	β-glucosidase	*Caldanaerobacter subterraneus subsp. tengcongensis* (strain DSM 15242 / JCM 11007 / NBRC 100824 / MB4) (*Thermoanaerobacter tengcongensis*)	449	66.1	1651
D3T6M2	β-glucosidase	*Thermoanaerobacter italicus* (strain DSM 9252 / Ab9)	447	66.4	1649
A0ABT9M0X5	β-glucosidase	*Thermoanaerobacter pentosaceus*	447	66.4	1649
I3VS74	β-glucosidase	*Thermoanaerobacterium saccharolyticum* (strain DSM 8691 / JW/SL-YS485)	446	63.4	1641
W9EEC0	β-glucosidase	*Thermoanaerobacterium aotearoense* SCUT27	446	63.4	1621
F6BL86	β-glucosidase	*Thermoanaerobacterium xylanolyticum* (strain ATCC 49914 / DSM 7097 / LX-11)	446	63.1	1627
C4L1S4	β-glucosidase	*Exiguobacterium* sp. (strain ATCC BAA-1283 / AT1b)	450	54.1	1347
B8CYA8	β-glucosidase	*Halothermothrix orenii* (strain H 168 / OCM 544 / DSM 9562)	451	53.9	1305
P26208	β-glucosidase	*Acetivibrio thermocellus* (strain ATCC 27405 / DSM 1237 / JCM 9322 / NBRC 103400 / NCIMB 10682 / NRRL B-4536 / VPI 7372) (*Clostridium thermocellum*)	448	50.7	1214
